# Identifying primary site of lung-limited Cancer of unknown primary based on relative gene expression orderings

**DOI:** 10.1186/s12885-019-5274-4

**Published:** 2019-01-14

**Authors:** Mengyao Li, Hongdong Li, Guini Hong, Zhongjie Tang, Guanghao Liu, Xiaofang Lin, Mingzhang Lin, Lishuang Qi, Zheng Guo

**Affiliations:** 1 Key Laboratory of Ministry of Education for Gastrointestinal Cancer, Department of Bioinformatics, Fujian Medical University, Fuzhou, 350001 China; 2 Department of Bioinformatics, Gannan Medical University, Ganzhou, 341000 China; 3 College of Bioinformatics Science and Technology, Harbin Medical University, Harbin, 150086 China; 4 Fujian Key Laboratory of Tumor Microbiology, Fujian Medical University, Fuzhou, 350001 China

**Keywords:** Cancer of unknown primary, Relative gene expression orderings, Metastasis, Lung cancer, Colorectal cancer

## Abstract

**Background:**

Precise diagnosis of the tissue origin for metastatic cancer of unknown primary (CUP) is essential for deciding the treatment scheme to improve patients’ prognoses, since the treatment for the metastases is the same as their primary counterparts. The purpose of this study is to identify a robust gene signature that can predict the origin for CUPs.

**Methods:**

The within-sample relative gene expression orderings (REOs) of gene pairs within individual samples, which are insensitive to experimental batch effects and data normalizations, were exploited for identifying the prediction signature.

**Results:**

Using gene expression profiles of the lung-limited metastatic colorectal cancer (LmCRC), we firstly showed that the within-sample REOs in lung metastases of colorectal cancer (CRC) samples were concordant with the REOs in primary CRC samples rather than with the REOs in primary lung cancer. Based on this phenomenon, we selected five gene pairs with consistent REOs in 498 primary CRC and reversely consistent REOs in 509 lung cancer samples, which were used as a signature for predicting primary sites of metastatic CRC based on the majority voting rule. Applying the signature to 654 primary CRC and 204 primary lung cancer samples collected from multiple datasets, the prediction accuracy reached 99.36%. This signature was also applied to 24 LmCRC samples collected from three datasets produced by different laboratories and the accuracy reached 100%, suggesting that the within-sample REOs in the primary site could reveal the original tissue of metastatic cancers.

**Conclusions:**

The result demonstrated that the signature based on within-sample REOs of five gene pairs could exactly and robustly identify the primary sites of CUPs.

## Background

Despite the recent advances in pathology investigations and imaging technology, the primary site remains unknown for about 3% of all the malignancies [1–3]. By definition, the cancer of unknown primary (CUP) is metastatic at diagnosis with unknown primary site, which indicates a high malignant degree with poor prognosis [4]. In clinical, the therapeutic strategy for CUPs often needs the recognition of primary sites, as the current clinical guidelines recommend the same or similar treatment scheme for metastases as their primary counterparts [5, 6].

Some investigators have tried to use gene expression profiling to predict the primary tumor sites for CUPs [7, 8]. For example, Greco *et al.* made use of a 92-gene molecular tumor profiling (MTP) assay to predict the primary tissues of CUPs, which showed an accuracy of 75% [8]. However, risk scores of such signatures rely on the absolute expression levels of genes, which could be affected by experimental batch effects [9]. Thus, such signatures often fail in independent samples [10–12]. It has been reported that the within-sample relative gene expression orderings (REOs) of gene pairs within individual samples are insensitive to experimental batch effects [10–12], invariant to monotonic data transformation [13, 14], and robust against partial RNA degradation [15] as well as sampling site uncertainty within a tumor tissue [16]. Based on these unique advantages, some classifiers based on REO signatures, such as TSP [10] and K-TSP [11], were proposed to identify transcriptional signatures for discriminating cancer subtypes [17–19], which obviate the need of data normalization for the discovery and validation datasets and thus can be applied to the individual level [17, 20, 21].

Colorectal cancer (CRC) is the third most frequent cancer worldwide, which accounts for approximately 10% of the global cancer burden [22, 23]. About 25% of the CRC patients present with metastases at diagnosis [24], of which liver and lung are the most frequent metastasis sites [25]. It has been reported that the lung metastases of CRC share high genomic concordance with the primary CRC [23]. Thus, we could hypothesize that the gene expression patterns of lung metastases of CRC would be more similar to the primary tumor on the susceptible primary organ than the metastatic organ. Here, using samples from the lung-limited metastatic colorectal cancer (LmCRC), we validated this hypothesis by comparing the stable REOs in LmCRC with the stable within-sample REOs in primary CRC and primary lung cancer, respectively. Then, we extracted a signature consisting of five gene pairs from primary CRC and lung cancer, and showed that this signature could predict all the LmCRC samples into the CRC-like group.

## Methods

### Data source and data preprocessing

The gene expression data analyzed in this study were downloaded from Gene Expression Omnibus (GEO, http://www.ncbi.nlm.nih.gov/geo/) [26]. Detailed information for each dataset was described in Table 1. Set1~Set3 denoted the gene expression profiles for primary CRC from three datasets respectively. Totally, there were 498 primary CRC samples. Set4~Set6 denoted the gene expression profiles for primary lung cancer from three datasets respectively. Totally, there were 509 primary lung cancer samples. The profiles were used to select the primary CRC and lung cancer characteristic REOs. Set7 denoted the gene expression profiles for LmCRC samples.

**Table 1:** The datasets analyzed in this study

Label	Dataset	Platform	Sample size	Ref (PMID)
Training sets				
Primary CRC				
Set1	GSE21510	GPL570	123	21270110
Set2	GSE14095	GPL570	189	21680303
Set3	GSE41258	GPL96	186	19359472
Primary lung cancer				
Set4	GSE31210	GPL570	226	22080568
Set5	GSE14814	GPL96	133	20823422
Set6	GSE43580	GPL570	150	23966112
Validation sets				
Primary CRC				
	GSE2138	GPL96	20	16247484
	GSE7208	GPL96	59	17638901
	GSE39582	GPL570	566	23700391
	GSE5364	GPL96	9	18636107
	GSE19249	GPL571	15	20522636
Primary lung cancer				
	GSE19804	GPL570	60	20802022
	GSE33532	GPL570	80	Michael Meister, *et al.*
	GSE18842	GPL570	46	20878980
	GSE5364	GPL96	18	18636107
	GSE19249	GPL571	7	20522636
Lung metastases of CRC				
Set7	GSE41258	GPL96	20	19359472
	GSE5851	GPL571	3	17664471
	GSE28702	GPL570	1	22095227

The raw data (.CEL files) for each dataset were downloaded from GEO and normalized by the robust multi-array average method (RMA) in the Bioconductor package [27–29] except GSE14095. Because the raw data were not provided, the normalized data provided by the authors were downloaded for GSE14095. The original platform annotation files obtained from GEO for each dataset were used to annotate the CloneIDs to GeneIDs.

### Detection of stable REO gene pairs

For a dataset, if gene *A* had a higher expression level than gene *B* in more than 95% samples, the gene pair (*A, B*) was defined as a stable REO gene pair. A concordance score was used to evaluate the reproducibility of stable REO gene pairs identified from two independent datasets. If two lists of stable REO gene pairs overlapped *k* gene pairs, of which *s* gene pairs had the same REO patterns, the concordance score will be calculated as *s/k* × 100%. The cumulative binomial distribution model was used to evaluate the probability of observing a concordance score of *s/k* × 100% by chance as follows:

1 $$ P=1-\sum \limits_{i=0}^{s-1}\left({}_i^k\right){\left({P}_e\right)}^i{\left(1-{P}_e\right)}^{k-1} $$

where *P*_*e*_ was the probability of a gene pair having the concordant relationship in the two datasets by chance (here, *P*_*e*_ = 0.5).

### Selection of predictive gene pairs as a candidate signature

If a stable REO gene pair (*A, B*) in class1 showed the reverse REO pattern (*B, A*) in class2, it was defined as a reversed gene pair between these two classes. Theoretically, according to the REOs of the reversed gene pairs, we could classify the samples in these two classes. The procedure for predictive signature selection was as follows:

Firstly, calculated the appearance frequency of each gene in reversed gene pairs.

Secondly, calculated the average rank difference score ΔavgR for each reversed gene pair, as described in formula (2):

2 $$ \Delta {avgR}_{ij}=\frac{\sum \limits_{n=1}^{N1}\left|{R}_{n,i}-{R}_{n,j}\right|+\sum \limits_{m=1}^{N2}\left|{R}_{m,i}-{R}_{m,j}\right|}{N1+N2} $$

Here N1 and N2 represented the number of profiles in class1 and class2, respectively. *R*_*n,i*_, *R*_*n,j*_, *R*_*m,i*_, and *R*_*m,j*_ represented the rank of gene *i* or *j* in the *n*-th and *m*-th profile of class1 and class2 respectively.

Thirdly, sorted the genes according to the appearance frequencies in the reverse order and selected one reversed pair with the maximum ΔavgR score for each gene.

At last, the top *n* gene pairs were selected as the candidate predictive signature.

### Anti-lung cancer drugs and protein-protein interaction data

The data of antitumor drugs and their target genes were collected from the DrugBank database (http://www.drugbank.ca/) [30], which contains 174 kinds of anticancer drugs approved by the U.S. Food and Drug Administration and 570 corresponding target genes. A total of 10 anti-lung cancer drugs and their 11 corresponding target genes were used in this study (Table 2).

**Table 2:** Anti-lung cancer drugs and their target genes

Drug ID	Drug	FDA	Target genes
DB00317	Gefitinib	approved	EGFR
DB00361	Vinorelbine	approved	TUBB
DB00642	Pemetrexed	approved	TYMS, ATIC, DHFR, GART
DB05390	INS 316	investigational	P2RY2
DB08865	Crizotinib	approved	ALK, MET
DB08916	Afatinib	approved	EGFR, ERBB2, ERBB4
DB09063	Ceritinib	approved	ALK
DB09330	Osimertinib	approved	EGFR
DB09559	Necitumumab	approved	EGFR
DB11363	Alectinib	approved	ALK

The human protein-protein interaction (PPI) data were constructed as previously described [31]. The PPI data were downloaded from Human Protein Reference Database (HPRD) [32] in November 2016.

## Results

### High REO concordance between lung metastases of CRC and primary CRC

To evaluate whether the REO patterns of lung metastases of CRC were similar to the primary CRC or lung cancer, a total of 498 primary CRC samples and 509 primary lung cancer samples were used (training set, Table 1).

First, gene pairs with stable REOs in more than 95% samples were identified and referred to as stable REO gene pairs. In the primary CRC Set1, Set2 and Set3, 156,893,727, 120,114,768 and 60,208,179 stable gene pairs were identified, respectively. Each two of the three lists of stable REO gene pairs showed significantly high concordances (Table 3), with a concordance score ranged from 91.3% (*P* <  2.20 × 10^− 16^) to 99.0% (*P* <  2.20 × 10^− 16^). There was a total of 35,220,621 stable REO gene pairs overlapped among these three datasets, which was denoted as primary CRC characteristic stable REO gene pairs. In primary lung cancer Set4, Set5 and Set6, 154,434,794, 53,985,252 and 140,533,599 stable REO gene pairs were identified, respectively (Table 3). A total of 31,739,263 stable REO gene pairs overlapped in all of these three datasets were denoted as primary lung cancer characteristic stable gene pairs.

**Table 3:** Concordance scores of stable REO gene pairs of primary CRC and primary lung cancer datasets

Dataset	Number of stable gene pairs	Number of overlaps	Concordance score	*p*-value
Primary CRC				
GSE21510	156,893,727	108,393,508	99.00%	< 2.20 × 10^−16^
GSE14095	120,114,768			
GSE21510	156,893,727	43,803,419	91.20%	< 2.20 × 10^−16^
GSE41258	60,208,179			
GSE14095	120,114,768	38,324,773	94.10%	< 2.20 × 10^− 16^
GSE41258	60,208,179			
Primary lung cancer				
GSE31210	154,434,794	35,809,034	86.00%	< 2.20 × 10^−16^
GSE14814	53,985,252			
GSE43580	140,533,599	128,549,112	99.60%	< 2.20 × 10^−16^
GSE31210	154,434,794			
GSE14814	53,985,252	33,941,889	88.10%	< 2.20 × 10^−16^
GSE43580	140,533,599			

**Fig. 1:**
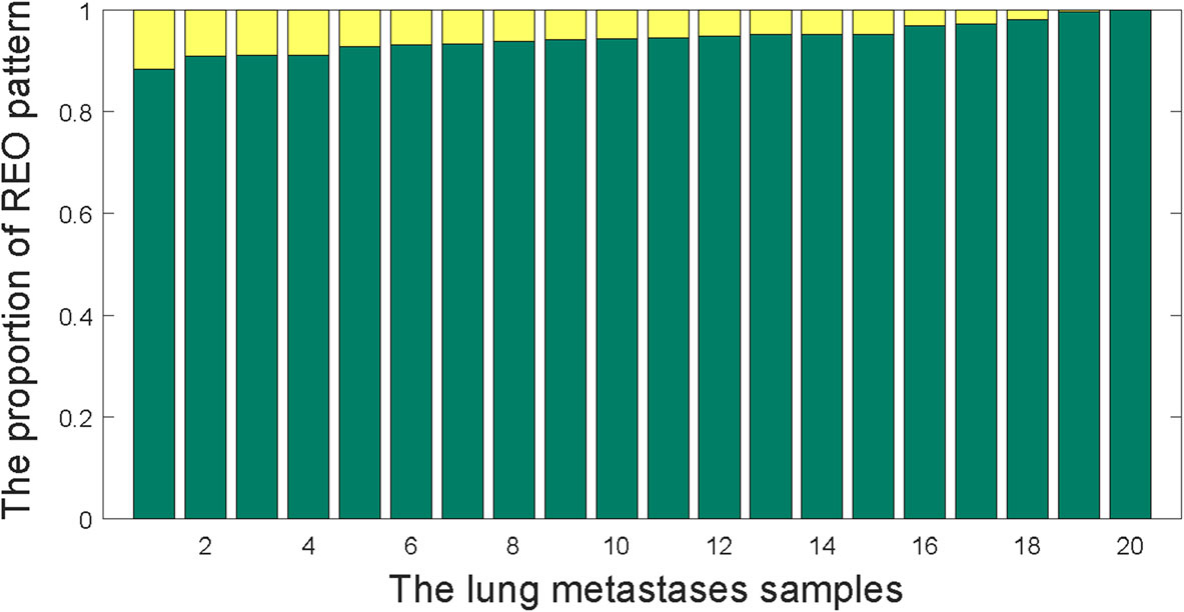
REO patterns of the 6599 gene pairs for each lung metastasis of CRC samples. The green bar stood for the proportion of REO pattern the same as that in primary CRC. The yellow bar stood for the proportion of REO pattern the same as that in primary lung cancer

Then, the characteristic stable REO gene pairs for primary CRC and lung cancer were compared to REO gene pairs identified for LmCRC samples. Between primary CRC and lung cancer, 6599 characteristic stable REO gene pairs showed the reverse REO patterns. These 6599 gene pairs (involving 4802 genes) were examined in each of the 20 LmCRC samples in Set7. In these 20 LmCRC samples, the REOs of the 6599 gene pairs were highly concordant with the primary CRC rather than with the primary lung cancer, which varied from the lowest concordance score of 88.36% to the highest concordance score of 99.89% (Fig. 1).

Collectively, these results indicated that, though with some characteristics of lung cancer, the lung metastases of CRC samples were more similar to the primary CRC. Therefore, the characteristic stable gene pairs of primary tumors could be applied to predict the primary tumor site of CUP.

### A robust signature for discriminating lung metastases of CRC from lung cancer

The 6599 gene pairs with reversal REO patterns between the primary CRC and primary lung cancer samples were used in developing the signature for discriminating the primary CRC and lung cancer. The candidate predictive signature was selected based on the appearance frequencies of genes in the reversed gene pairs between primary CRC and lung cancer and the average rank difference score ΔavgR of each gene pair, as described in Methods. Then, sequentially took odd numbered gene pairs (i.e. 1, 3, 5, ... gene pairs) from the candidate gene pair list to classify primary CRC and lung cancer samples by the majority voting rules: if more than half of the REOs of the gene pairs in a sample were consistent with the candidate signature gene pairs, the sample would be predicted into CRC-like group, otherwise, the sample would be predicted into the lung cancer-like group. The classification accuracy was 99.36% when five gene pairs were taken, and kept unchanged at 99.36% from five gene pairs to 43 gene pairs. Interestingly, the gene SLC34A2 was included in all the 5 gene pairs (Table 4). This gene was reported to play an essential role in the tumorigenesis and progression of non-small cell lung cancer [33] and other pneumonosis such as pulmonary alveolar microlithiasis [34].

**Table 4:** Top five genes with highest appearance frequencies and their gene pairs with maximum ΔavgR

Gene Symbol	Appearance Frequency	Gene Pair Symbol^a^	ΔavgR
GUCY2C	1969	GUCY2C, SLC34A2	10,298.34705
CDH17	1322	CDH17, SLC34A2	10,273.16384
FABP1	485	FABP1, SLC34A2	10,001.53088
SLC34A2	474	KRT20, SLC34A2	10,657.31487
USH1C	270	USH1C, SLC34A2	9020.681651

^a^The former genes had higher expression levels than the latter genes in the CRC

The prediction capacity of the signature was further tested in an independent dataset comprising 654 primary CRC and 204 primary lung cancer samples collected from seven datasets (Table 1). The result showed that 99.54% of the 654 primary CRC samples were correctly predicted into the CRC group and 99.51% of the 204 primary lung cancer samples were correctly predicted, reaching an average prediction accuracy of 99.53%. This result suggested that the signature had a robust discriminating capability to distinguish the primary CRC and lung cancer samples.

Using the signature to predict the 20 LmCRC samples collected from 3 datasets (Table 1), the result showed that all these samples were correctly classified to the CRC group. The accurate prediction indicated that the REO patterns in primary CRC and primary lung cancer could be applied to identify the tissue of origin for pulmonary tumor. There were another three lung metastases of CRC samples in GSE5851 and one in GSE28702, which were also predicted to the CRC group, with the prediction accuracy of 100%.

### Lung cancer characteristics of lung metastases of CRC

**Fig. 2:**
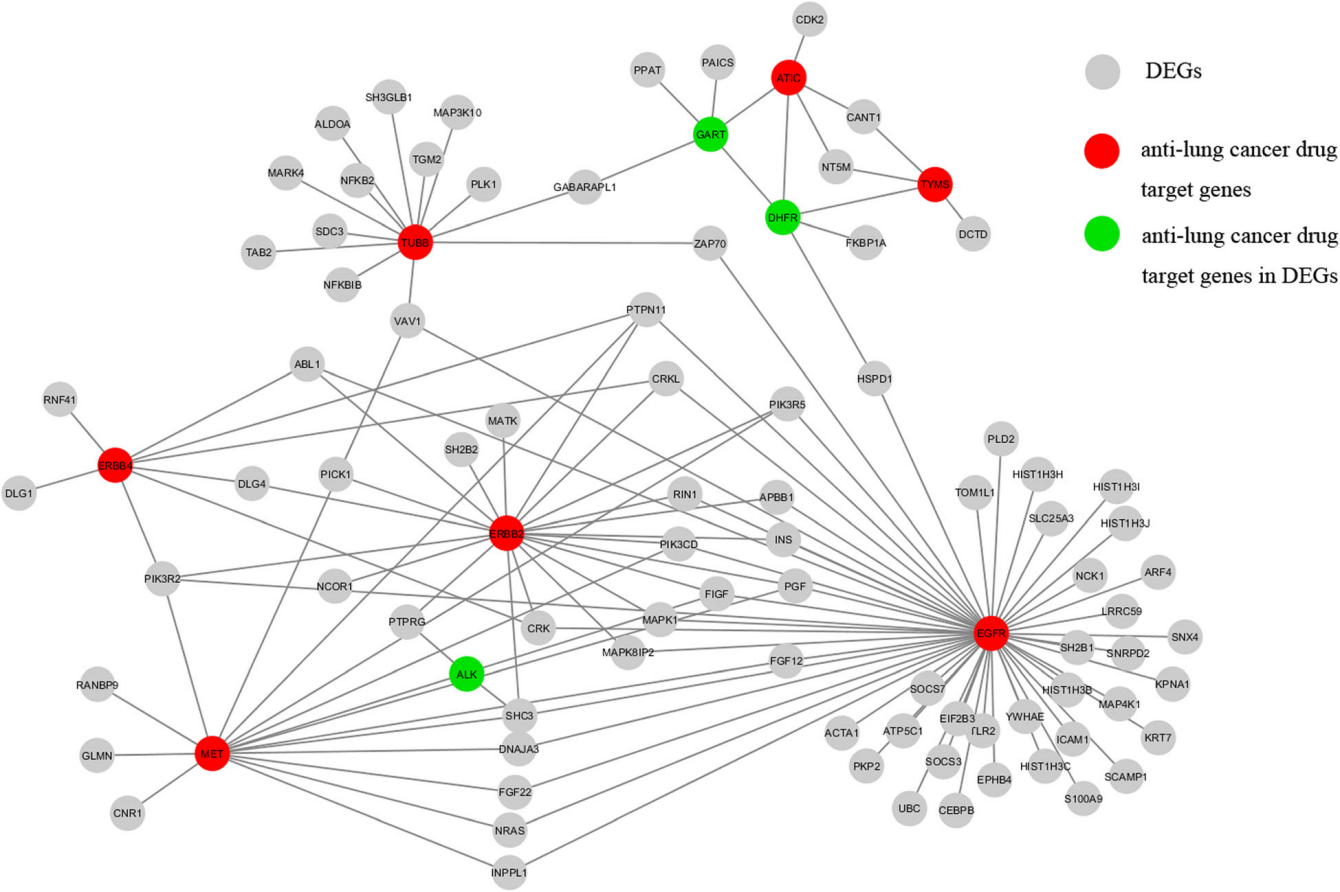
The PPI links between DEGs and anti-lung cancer drug target genes

A total of 2034 differentially expressed genes (DEGs) were distinguished between the primary CRC and lung metastases of CRC in GSE41258 by the Student’s *t*-test with false discovery rate (FDR) less than 1%. In the PPI network, 90.91% (10) of the 11 anti-lung cancer drugs target genes had direct PPI links with at least 119 DEGs (Fig. 2). Especially, three anti-lung cancer drugs target genes (DHFR, GART and ALK) also presented in the DEGs. The DEGs centrally had direct interaction with EGFR, ERBB2, ERBB4, MET and TUBB, which suggested that the divergence between the primary CRC and the metastases of CRC was related to lung cancer. Furthermore, the direct interaction with anti-lung cancer drug target genes indicated these target genes could also be regarded as the CRC lung metastases treatment target genes, and their corresponding drugs, including Osimertinib, Necitumumab, Gefitinib, Afatinib, Osimertinib, Necitumumab, Crizotinib and Vinorelbine could be considered to be included in the LmCRC regimen.

## Discussion

Up to now, there are massive data of primary tumors in the public databases, whereas the data for metastases are rare. As the REO-based signature is insensitive to experimental batch effects, we could obviate the need for data normalization for the discovery and validation datasets. Especially, because the REO-based signature obviates the need for data normalization, it can be applied at the individual level [17, 20, 21].

The linear progression model, where the accumulated genetic alterations in the primary tumor could lead to metastases, is generally accepted in researches on cancer progression [23]. In the linear progression model, the metastases acquire most traits of the primary site. Accordingly, the treatment for the metastases is the same or similar to their primary counterparts. Therefore, to precisely identify whether a lesion is the primary tumor or metastasis from other cancerous organs is essential for tailoring the regimen. The liver- and lung-limited metastases are the most frequent migrating targets of CRC [25]. In this study, taking the LmCRC as an example, we showed a high concordance rate between the REOs within lung metastases and their primary CRC tissues other than the primary lung cancer tissues, which provided a direct evidence for the linear progression model.

On the other hand, there might be some differences between the lung metastases and primary CRC. By using the Student’s *t*-test with FDR < 0.01, 2034 DEGs were detected between the primary CRC and lung metastases samples in GSE41258, among which 274 genes were also detected as DEGs between the primary CRC and primary lung cancer samples in GSE19249. Then, we made use of the normal colorectal tissue and lung tissue samples to further explore whether these 274 DEGs might exhibit lung tissue-specific characteristics. The result showed that 52 of the 274 DEGs were DEGs between the normal colorectal tissue and lung tissue samples (Student’s *t*-test with FDR < 0.01). These results indicated that the lung metastases of CRC might possess some characteristics of the host organ, which needs to be further confirmed by analyzing microdissected samples of lung metastases of CRC to eliminate the possible confounding influence of residual lung tissues in the LmCRC samples. Finally, a PPI network analysis was conducted for DEGs between the primary CRC and their lung counterparts. As shown in Fig. 2, three DEGs (DHFR, GART and ALK) also played roles as anti-lung cancer drugs target genes. As the treatment for lung-limited metastases of CRC was curative resection accompanied with the regimen for CRC [35, 36], the analysis indicated that some lung cancer drugs could be recommended for LmCRC patients, which deserves further study for tailoring the treatment regimen for the LmCRC patients.

## Conclusions

The REOs-based signature could identify the primary tissue of LmCRC with an accuracy of 100%. The within-sample REOs in primary sites could be a powerful approach for predicting the origin tissues of CUPs.

## Abbreviation

CRC: Colorectal cancer
CUP: Cancer of unknown primary
DEGs: Differentially expressed genes
FDR: False discovery rate
GEO: Gene Expression Omnibus
LmCRC: Lung-limited metastatic colorectal cancer
MTP: Molecular tumor profiling
PPI: Protein-protein interaction
REOs: Relative gene expression orderings
RMA: Robust multi-array average
